# Effect of aflibercept in insufficient responders to prior anti-VEGF therapy in neovascular AMD

**DOI:** 10.1007/s00417-014-2589-3

**Published:** 2014-03-11

**Authors:** Heidi Fassnacht-Riederle, Matthias Becker, Nicole Graf, Stephan Michels

**Affiliations:** 1 Department of Ophthalmology, City Hospital Triemli, Birmensdorferstrasse 497, 8063 Zurich, Switzerland; 2 Department of Ophthalmology, University of Heidelberg, Heidelberg, Germany; 3 Graf Biostatistics, Winterthur, Switzerland; 4 University of Zurich, Zurich, Switzerland

**Keywords:** Age-related macular degeneration, Aflibercept, Ranibizumab, Bevacizumab, Tolerance, Tachyphylaxis

## Abstract

**Purpose:**

Evaluation of three aflibercept injections at 4-week intervals in patients with neovascular AMD showing an “insufficient anatomic response” to prior anti-VEGF therapy with ranibizumab or bevacizumab.

**Methods:**

The retrospective analysis included 96 eyes that had received at least three intravitreal 0.5 mg ranibizumab or 1.25 mg bevacizumab injections over a period of no more than 4 months prior to switching to aflibercept. In addition, the selected eyes had to have evidence of persisting or increasing sub- or intraretinal fluid, observed in optical coherence tomography (OCT). All patients received a loading dose of three intravitreal 2 mg aflibercept injections at 4-week intervals. Evaluation included central retinal thickness (CRT) and maximum pigment epithelium (PED) height measured by spectral domain OCT and best-corrected visual acuity (BCVA) prior to the switch of therapy and 4 weeks after the third aflibercept injection.

**Results:**

A significant reduction of mean CRT (−39 μm; *p* < 0.001) and maximum PED height (−46 μm; *p* < 0.001) as found 4 weeks after the third aflibercept injection. Eighty-two out of 96 eyes (85 %) had a PED just prior to switching to aflibercept. There was an improvement in BCVA of 1.9 letters 4 weeks after the last aflibercept injection; the vision gain, however, did not reach statistical significance (*p* = 0.061). The further analysis did not show any correlation of the change in CRT, maximum PED, and BCVA with the number of prior anti-VEGF treatments.

**Conclusion:**

Retinal edema and PEDs regressed significantly after switching to aflibercept in patients insufficiently responding to prior therapy with ranibizumab or bevacizumab. No correlation could be found with regard to the number of prior treatments.

## Introduction

The introduction of intravitreal pan anti-vascular endothelial growth factor (VEGF) blockade for neovascular age-related macular degeneration (AMD) in the last decade has had a significant beneficial impact on patients with neovascular AMD [1–3]. However, information on long-term functional outcomes on anti-VEGF therapy in neovascular AMD are sparse and limited by decreasing number of patients in follow-up. A 7-year follow-up study on ranibizumab indicates a mean vision loss of more than 8 letters and 34 % of patients having a loss of at least 3 lines in vision at the final visit [4]. The vision loss in long-term follow-up is probably multifactorial. It includes natural progression of the underlying non-neovascular AMD, too few anti-VEGF treatments despite a still active disease, and the loss of treatment effectiveness over time [5].

Several studies have shown that a reduced anatomic response can be found for ranibizumab and bevacizumab in neovascular AMD over time [6, 7]. The terms used for this finding, range from tolerance or tachyphylaxis to resistance [5]. It is assumed that persistent intra- or subretinal edema will lead to degenerative changes and subsequent vision loss. Various reasons, including up-regulation of alternative pathways, autoantibodies, and impairment of the retinal pigment epithelium (RPE) have been implicated [8]. To overcome the “insufficient response”, several treatment strategies have been used. Ranibizumab and bevacizumab have been tried in exchange, showing some anatomic response in most cases, but the functional success has been limited [9]. Short-term follow-up, in cases of reduced anatomic response (at least some regression showed) could indicate that more frequent retreatments might be useful to reduce edema. However, retreatments that are more frequent than every 4 weeks have significant implications for patients, including higher ocular risks and an additional financial burden as such retreatments are often not reimbursed by health care systems. Increasing the injected drug dose (mostly doubling) appears as an alternative, but extends biological activity by only one half-life time [10]. Moreover, the injection of 0.1 ml leads to a higher intraocular pressure (IOP) post-injection than the usually used 0.05 ml. So far, there is limited data on combination therapies with steroids or photodynamic therapy [7].

Aflibercept is a new VEGF inhibitor that differs from ranibizumab and bevacizumab by a higher affinity to VEGF. By entrapping the VEGF dimer, aflibercept avoids two drug molecules binding to one VEGF dimer. In addition, aflibercept blocks the placental growth factor (PlGF). The presented study aimed to evaluate eyes anatomically “insufficiently responding” to prior anti-VEGF therapy with ranibizumab and bevacizumab in a European population, with special focus on the prior treatment history.

## Methods

The presented study is a retrospective analysis of patients treated with aflibercept for neovascular AMD at the department of Ophthalmology at the City Hospital Triemli in Zurich. The study adhered to the Declaration of Helsinki, and obtained exemption from full review by the local ethics committee. Records of patients treated for neovascular AMD with a first intravitreal aflibercept between November 2012 to May 2013 were reviewed based on defined inclusion criteria. To select patients insufficiently responding to prior therapy, a two-step selection process was used. The first criterion required that the affected eye had received at least three intravitreal 0.5 mg ranibizumab or 1.25 mg bevacizumab injections over a period of no more than 4 months prior to switching to aflibercept. Secondly, the selected eyes had to have evidence of insufficient anatomic response to prior therapy, defined as any persisting or increasing sub- or intraretinal fluid observed in spectral-domain optical coherence tomography (OCT) using the Heidelberg Spectralis® system (Heidelberg Engineering, Heidelberg Germany). All 19 standard scans (setting: 512 A scans, 20° × 15°) were compared between scans taken just prior to the first of the last three injections with ranibizumab or bevacizumab therapy and the scans taken just prior to initiation of aflibercept therapy. Evidence of any persisting sub- or intraretinal fluid in any of the 19 scans was considered an insufficient response. OCT scans were compared based on “follow-up” mode of the eye-tracking-assisted Heidelberg Spectralis® system (AutoRescan), allowing precise comparison. In general, all eyes initiated with aflibercept were planned to receive three intravitreal 2 mg aflibercept injections at 4-week intervals, with a final visit 4 weeks (±1 week) after the last injection, according to the Swiss label of aflibercept. The retrospective analysis included only eyes that effectively obtained this treatment.

We recorded demographic data, the total number of intravitreal pretreatments with ranibizumab and/or bevacizumab, the time since initiation of any anti-VEGF therapy, the anti-VEGF drug used prior to changing to aflibercept, the interval between the last intravitreal pretreatment with ranibizumab or bevacizumab, and the first aflibercept injection.

The primary outcome was the change in central retinal thickness (CRT) measured by OCT 4 weeks after the third aflibercept injection, compared to the time of change in therapy.

Secondary outcome measures were the change in maximum height of a pigment epithelial detachment (PED) — if present — as well as the change in best-corrected visual acuity (BCVA) using Snellen visual acuity. The change in maximum height of the PED was measured via the software’s ruler tool in μm on the OCT image. For visual acuity analysis, Snellen readings were transferred to ETDRS letters according to the publication by Gregori et al. [11]. All measurements were conducted just prior to the first aflibercept injection and 4 weeks (±1 week) after the third aflibercept injection.

Furthermore, we analysed whether the changes in CRT, PED, or BCVA correlated with the total number of pretreatments (i.e. using ranibizumab and/or bevacizumab), or with the anti-VEGF drug used immediately prior to the change in therapy. Finally, the intraocular pressure (IOP) immediately prior to the change in therapy was compared to the IOP at the final visit. Adverse events were recorded.

All statistical analyses were done using SPSS® Version 20. For inferential statistics, non-parametric tests were performed for all analyses, since distributions were not normal. Differences were calculated by subtracting pretreatment data from posttreatment data. Dependent samples were tested with the exact Wilcoxon signed rank test, and independent samples with the exact Mann–Whitney U test. Spearman’s rho was calculated for all correlation analyses. Statistical significance was defined as *p* < 0.05.

## Results

A total of 109 eyes with neovascular AMD and pretreatment with ranibizumab and/or bevacizumab fulfilled the definition of an insufficient treatment effect, and were switched to therapy with aflibercept. Thirteen eyes were not included in the analysis because they had not received the three planned injections, or because they did not fulfill the timing schedule. Thus, a total of 96 eyes of 88 patients were analyzed.

The mean age of the involved patients was 78.9 years. Fifty-three of the 96 treated eyes were of female patients. The majority of eyes were treated only with ranibizumab (*n* = 64), four were treated only with bevacizumab, and 28 eyes have been switched from bevacizumab to ranibizumab or vice versa.

On average, a total of 26.9 anti-VEGF pretreatments with ranibizumab and/or bevacizumab were administered prior to changing therapy to aflibercept. Of these pretreatments, the mean number of injections was 24.8 for ranibizumab and 9.2 for bevacizumab.

The first anti-VEGF treatment was given on average 35 months prior to switch of therapy, and the mean retreatment interval since initiation of any anti-VEGF therapy was 1.3 months.

**Fig. 1:**
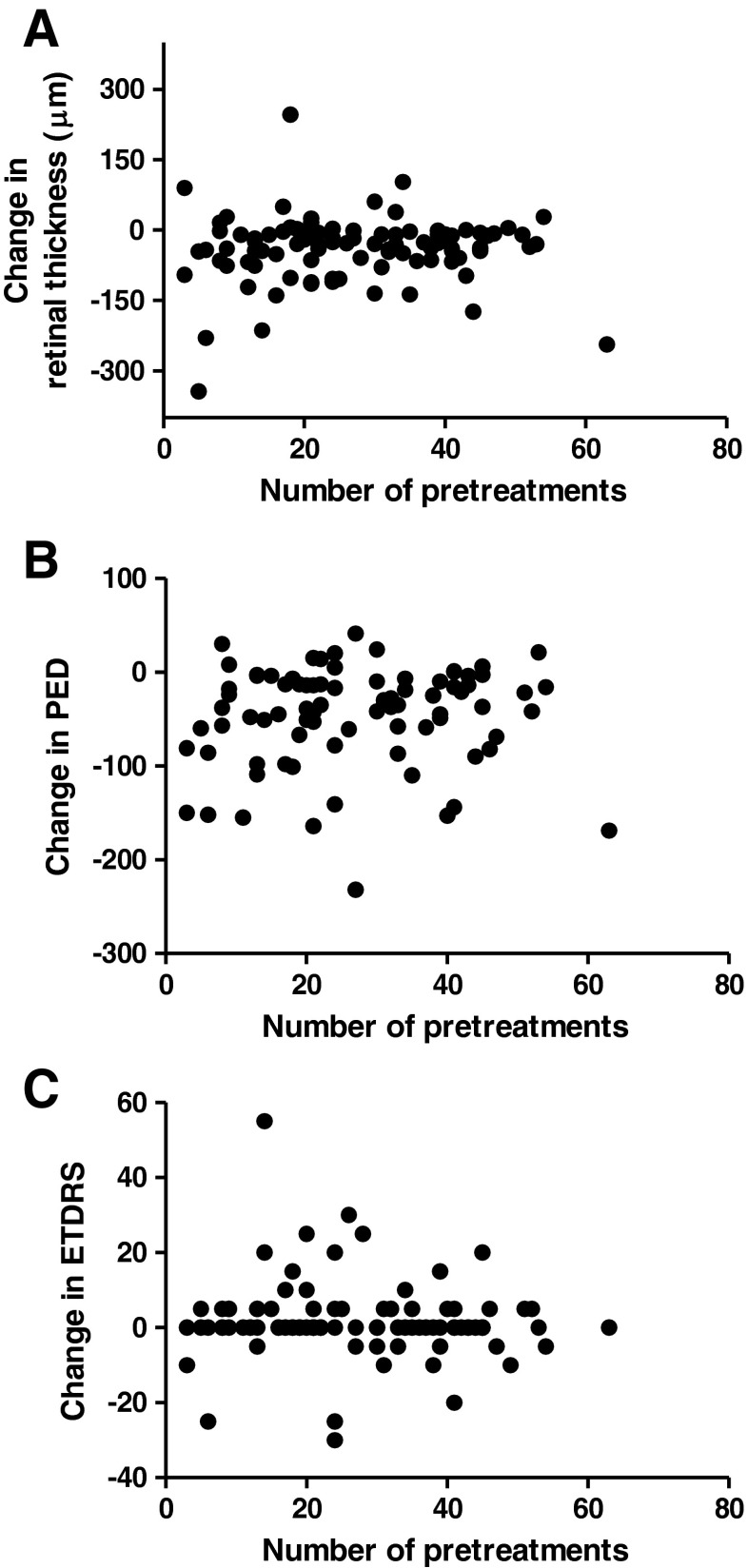
**a** Change in central retinal thickness 4 weeks after the third treatment with aflibercept does not correlate with the total number of prior anti-VEGF treatments with ranibizumab or bevacizumab (Spearman’s rho 0.069, *p* = 0.507)**. b** Change in maximum PED height 4 weeks after the third treatment with aflibercept does not correlate with the total number of prior anti-VEGF treatments with ranibizumab or bevacizumab (Spearman’s rho 0.121, *p* = 0.278). **c** Change in BCVA 4 weeks after the third treatment with aflibercept does not correlate with the total number of prior anti-VEGF treatments with ranibizumab or bevacizumab (Spearman’s rho −0.107, *p* = 0.298)

The mean retinal thickness was 337 μm (median 322 μm) before treatment with aflibercept compared to 298 μm following three aflibercept injections (median 277 μm). Thus, with regard to the primary outcome, we measured a significant mean reduction in retinal thickness of 39 μm (*p* < 0.001) observed 4 weeks after the third aflibercept injection. Patients with ranibizumab or bevacizumab as last pretreatment preceding aflibercept injection did not differ significantly with regard to retinal thickness before and after treatment (*p* = 0.739). No correlation between the total number of pretreatments (i.e. ranibizumab and/or bevacizumab) and the change in retinal thickness could be found (Spearman’s rho = 0.069, *p* = 0.507). Figure 1a shows the point by point findings with regard to change in CRT and number of pretreatments.

There were 83 eyes with a PED at the first assessment, of which 82 eyes had valid data for PED at the follow-up visit. Thus, 82 eyes were analyzed for PED.

The maximum height of PED decreased significantly by 46 μm (*p* < 0.001) from a mean of 241 μm (median 208 μm) prior to aflibercept injection to 195 μm (median 162 μm) following the third aflibercept injection. The type of most recent pretreatment (ranibizumab or bevacizumab) immediately prior to changing therapy did not have a significant influence on maximum PED (*p* = 0.910). No correlation between the total number of pretreatments (i.e. ranibizumab and/or bevacizumab) and the change in maximum height of the PED could be observed (Spearman’s rho = 0.121, *p * = 0.278) as shown in the point by point presentation of “number of prior treatments” and “change in maximum PED height” in Fig. 1b.

Mean BCVA was 61.6 letters prior to switching to aflibercept. Four weeks after the third aflibercept treatment, there was an increase of 1.9 letters in visual acuity, which was just below significance level (*p* = 0.061). The type of the most recent pretreatment (ranibizumab or bevacizumab) did not have a significant influence on the change in BCVA (*p* = 0.986).

**Fig. 2:**
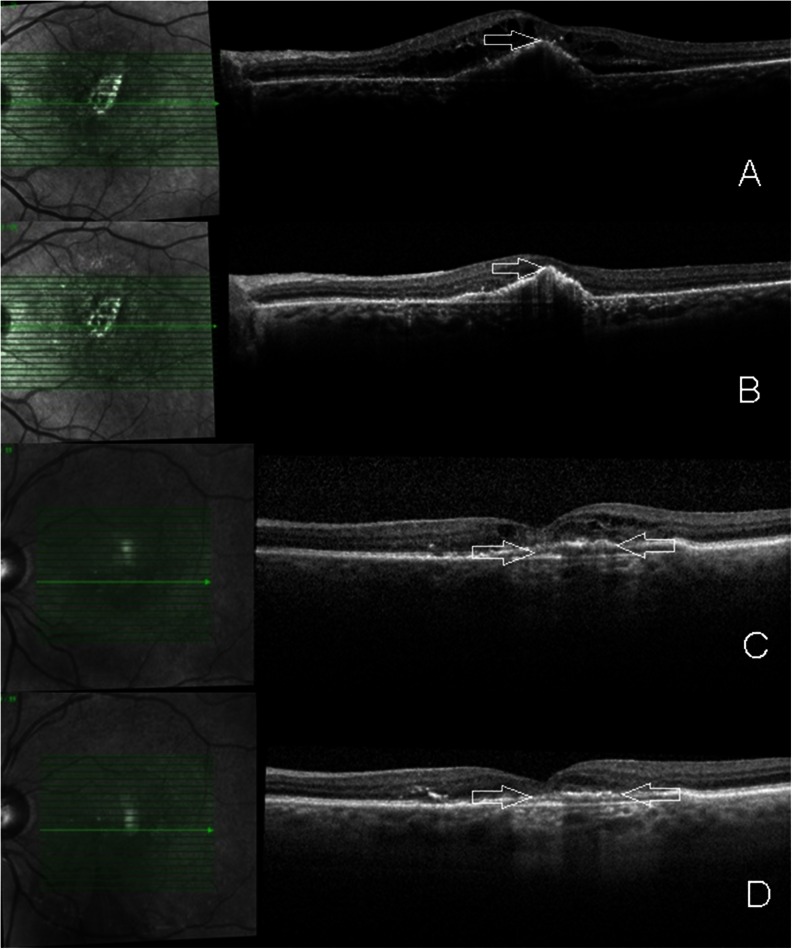
Spectral-domain optical coherence tomography images through the foveal region prior to (**a**/**c**) and after (**b**/**d**) three aflibercept injections in two cases with persistent edema with prior ranibizumab therapy. Despite the resolution of intraretinal/subretinal fluid and a slight regression of the PED, the two eyes lost more than 3 lines in vision. Both eyes had some degree of pre-existing subfoveal RPE changes (*arrows* in **a**/**c**). Post aflibercept treatment, RPE atrophy appears more evident (*arrows* in **b**/**d**). However progression of preexisting atrophy cannot be concluded due to a potential “masquerading” effect of edema present prior to switch of treatment

Similarly to CRT and maximum PED height, no correlation could be observed between the total number of pretreatments and the change in BCVA (Sperman’s rho −0.107, *p* = 0.298). Figure 1c shows each BCVA outcome in association with the number of pretreatments. Four eyes had a BCVA loss of more than three lines. All four eyes showed an anatomic response (regression of intra- and subretinal fluid). In one eye, the vision loss was transient, most likely due to a viral conjunctivitis. In the other three eyes, vision loss was persistent and attributed to atrophy of the RPE and/or fibrotic changes. Figure 2 shows the central OCT scans of two of the eyes with persistent vision loss of more than 3 lines.

No severe ocular or systemic adverse events occurred within the 3-month follow-up period. Despite a high percentage of eyes with PEDs, no new tear of the RPE was seen within the 3-month follow-up period. None of the eyes showed a non-spontaneously resolving intraocular inflammation under treatment with aflibercept. The mean intraocular pressure showed a mild, but significant decrease from 15.1 mmHg prior to aflibercept to 13.7 mmHg after the third aflibercept injection (*p* < 0.001).

## Discussion

Switching anti-VEGF therapy to aflibercept in patients with neovascular AMD who were showing insufficient anatomic response with ranibizumab or bevacizumab was accompanied by a reduction of central retinal thickness and maximum PED height, in a short-term follow-up. An improvement in visual acuity by 1.8 letters did not reach statistical significance. Our data are in line with other studies evaluating aflibercept in cases of neovascular AMD showing insufficient anatomic response to previous anti-VEGF therapy [12–17]. There is consistency with regard to an anatomical improvement. However, not all studies show a significant gain in visual acuity. Since visual acuity was not the primary outcome of our study, intra- or subretinal fluid in a subfoveal location was not an inclusion criterion, which may explain the non-significant improvement in visual acuity in our study. Other factors potentially providing different functional results might include different lesion types, variations in the definition of “insufficient response” to previous therapy, and the type of treatment regimen used with aflibercept.

Most studies with limited or no vision gain explain their results with the long-standing disease. However, the present study evaluated this aspect, but could not find a correlation of increased anatomical or functional outcome with regard to fewer pretreatments, as shown in Fig. 1a-c. This would indicate that at least with regard to anatomical outcomes, a switch in therapy would make sense. This is independent of the number of pretreatments, if “insufficient anatomic response” (as defined in our analysis) is seen with other anti-VEGF agents. Reduced or absent edema could prevent vision loss in further follow-up, which remains to be proven in studies with long-term follow-up. The characteristics of the selected patient population prior to starting aflibercept are worth mentioning. Visual acuity was relatively good with 61.6 letters (20/63^+^) prior to switching to aflibercept, despite a mean of 26.9 prior anti-VEGF injections. These were performed on average every 1.3 months. This patient population had initially lost 3.1 letters over a mean of 35.0 months with ranibizumab and/or bevacizumab. This indicates that despite no under-treatment (>9 injections per year on average), the selected patient population did not perform well under ranibizumab and bevacizumab. It also could imply that some persistent fluid impairs functional outcomes.

Formerly, PEDs without significant growth were not considered as an indication for treatment in the case of absence of intra- or subretinal fluid in neovascular AMD under prior anti-VEGF treatment. The significant effect on PEDs after the switch to aflibercept is remarkable, especially when taking into consideration that many PEDs did not respond to previous therapy [16]. The questions therefore arise, whether we should treat PEDs more aggressively with the new anti-VEGF treatment option, and whether VEGF inhibition is related to this effect. Despite a clear regression of PEDs under aflibercept treatment, no new tear of the RPE occurred in our study. Since an effect on PEDs was seen, the high number of pretreatments might only be a partial explanation for the absence of new RPE tears in this population.

It is unclear why all studies find reduced edema following a switch to aflibercept. Theoretically, all VEGF should be bound by intensive anti-VEGF therapy. Better retinal penetration appears rather unlikely, especially when compared to the much smaller ranibizumab molecule. The additional binding of the placental growth factor (PlGF) by aflibercept, its higher binding affinity to VEGF compared to other VEGF inhibitors, or the development of auto-antibodies to prior anti-VEGF therapy, could serve as potential explanations [8].

In conclusion, the switch to aflibercept in neovascular AMD “insufficiently responding” to prior anti-VEGF therapy clearly has a beneficial anatomical effect, even on long-persisting PEDs, and is worth a try independent of the number of pretreatments.
